# Prognostic significance of the stress hyperglycemia ratio in critically ill patients

**DOI:** 10.1186/s12933-023-02005-0

**Published:** 2023-10-13

**Authors:** Le Li, Minghao Zhao, Zhuxin Zhang, Likun Zhou, Zhenhao Zhang, Yulong Xiong, Zhao Hu, Yan Yao

**Affiliations:** Chinese Academy of Medical Sciences, Peking Union Medical College, National Center for Cardiovascular Diseases, Fu Wai Hospital, Beijing, 100037 China

**Keywords:** Stress hyperglycemia ratio, Critical ill, Outcomes, Restricted cubic spline

## Abstract

**Background:**

The stress hyperglycemia ratio (SHR) has demonstrated a noteworthy association with unfavorable cardiovascular clinical outcomes and heightened in-hospital mortality. Nonetheless, this relationship in critically ill patients remains uncertain. This study aims to elucidate the correlation between SHR and patient prognosis within the critical care setting.

**Methods:**

A total of 8978 patients admitted in intensive care unit (ICU) were included in this study. We categorized SHR into uniform groups and assessed its relationship with mortality using logistic or Cox regression analysis. Additionally, we employed the restricted cubic spline (RCS) analysis method to further evaluate the correlation between SHR as a continuous variable and mortality. The outcomes of interest in this study were in-hospital and 1-year all-cause mortality.

**Results:**

In this investigation, a total of 825 (9.2%) patients experienced in-hospital mortality, while 3,130 (34.9%) individuals died within the 1-year follow-up period. After adjusting for confounding variables, we identified a U-shaped correlation between SHR and both in-hospital and 1-year mortality. Specifically, within the SHR range of 0.75–0.99, the incidence of adverse events was minimized. For each 0.25 increase in the SHR level within this range, the risk of in-hospital mortality rose by 1.34-fold (odds ratio [OR]: 1.34, 95% CI: 1.25–1.44), while a 0.25 decrease in SHR within 0.75–0.99 range increased risk by 1.38-fold (OR: 1.38, 95% CI: 1.10–1.75).

**Conclusion:**

There was a U-shaped association between SHR and short- and long-term mortality in critical ill patients, and the inflection point of SHR for poor prognosis was identified at an SHR value of 0.96.

**Supplementary Information:**

The online version contains supplementary material available at 10.1186/s12933-023-02005-0.

## Introduction

Stress hyperglycemia, characterized by elevated admission blood glucose (ABG), is a common occurrence among patients grappling with trauma and critical illness [1]. Previous study reported that nearly 10% patients admitted to intensive care unit (ICU) suffered from stress hyperglycemia [2]. In the context of acute illness, stress hyperglycemia represents an evolutionarily preserved adaptive response aimed at enhancing the host’s chances of survival; nevertheless, it may directly contribute to adverse outcomes by triggering mechanisms such as the induction of endothelial dysfunction and oxidative stress [3]. Numerous studies have unequivocally established a clear correlation between stress hyperglycemia and adverse outcomes, including increased mortality, heightened morbidity, prolonged hospitalizations, heightened susceptibility to infections, and an overall rise in complications within ICU settings. [4–6].

Nonetheless, the ABG level is impacted not solely by acute stress but also by underlying chronic glycemic conditions, thereby constraining its capacity to accurately discern a genuine acute glycemic surge. In this setting, stress hyperglycemia ratio (SHR), which is adjusted by the average glycemic status, is proposed to evaluate the actual blood glucose status [7]. Although previous studies have reported that SHR is an independent risk factor for mortality in certain population [8–11], the relationship between SHR and prognosis among critically ill patients remains incompletely elucidated. In this study, our objective was to explore the prognostic relationship between SHR and mortality within a substantial, critical care cohort.

## Method

### Study design

In this investigation, a retrospective cohort study was undertaken utilizing the comprehensive Medical Information Mart for Intensive Care IV (MIMIC-IV, version 2.0), a robust US-based database. This repository encompasses an extensive array of health-related data originating from 76,943 unique ICU admissions, encompassing 53,150 distinct patients who underwent critical care at the Beth Israel Deaconess Medical Center over the time span from 2008 to 2019 [12]. Within this context, one of the authors, Le Li, received proper authorization to access the database, and was attributed a designated Record ID of 35,965,741. To ensure the utmost protection of patient privacy, all personally identifiable information underwent meticulous de-identification procedures. It’s worth noting that since our study is centered around the analysis of a third-party anonymized and openly accessible database, which had already received the approval of an institutional review board (IRB), the IRB review process at our own institution was appropriately determined to be exempted.

### Cohort selection

**Fig. 1:**
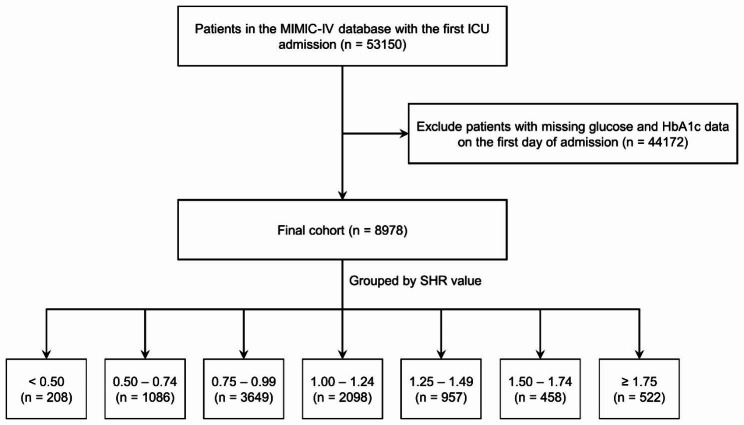
Flow chart. ICU: intensive care unit; SHR: stress hyperglycemia ratio

Our study encompassed patients who were admitted to the ICU for the first time, and those who did not undergo glycosylated hemoglobin A1c (HbA1c) or glucose tests within the initial 24 h after admission were excluded from the analysis. It is important to underscore that within the MIMIC-IV 2.0 database, individuals aged below 18 years were automatically excluded. Following these criteria, a total of 8,978 patients were selected for inclusion in the ICU analysis (Fig. 1). Based on the SHR levels, the patient cohort was divided into seven distinct groups, with intervals of 0.25, spanning from < 0.50 to ≥ 1.75.

### Variable extraction

The variables collected in this study include patient demographics (age, sex, weight), common comorbidities (diabetes mellitus [DM], hypertension, myocardial infarction [MI], chronic kidney disease [CKD] et al.), survival outcomes (in-hospital mortality and 1-year mortality), severity score (SAPS-II, LODS, et al.), laboratory parameters (white blood cell [WBC], hemoglobin [HGB], serum creatinine [SCr], blood urea nitrogen [BUN]), medication and interventions (insulin, vasopressor, diuretics and mechanical ventilation [MV]), and other relevant variables. The SHR was calculated as follows:

$$\text{S}\text{H}\text{R}=\text{A}\text{B}\text{G} (\text{m}\text{g}/\text{d}\text{L}) / (28.7\times \text{H}\text{b}\text{A}1\text{c} (\text{\%})-46.7)$$

Glucose and HbA1c values were using the first record after ICU admission. All comorbidities were identified based on ICD-9 or ICD-10 codes. Information regarding hospitalization within the initial 24 h following ICU admission was meticulously extracted from the MIMIC-IV database through the utilization of PostgreSQL (version 14.0). This study adhered to the Strengthening the Reporting of Observational Studies in Epidemiology (STROBE) guidelines for observational studies [13].

### Outcomes

The primary endpoint was in-hospital all-cause mortality, with 1-year all-cause mortality serving as the secondary endpoint. However, it’s crucial to highlight that the MIMIC-IV database restricts access to death dates beyond one year from the last hospital discharge. Consequently, the database does not facilitate insights into patient mortality beyond the one-year timeframe, which restricts the utilization of MIMIC-IV data for inferences related to deaths occurring beyond that period.

### Statistical analysis

All analyses were performed using R software (version 4.1.0), and 2-side P < 0.05 was considered statistically significant. Categorical variables were presented as proportions, while continuous variables were depicted as either mean (standard deviation, SD) or median (interquartile range, IQR). The Wilcoxon test was employed to compare continuous variables, while categorical variables were subjected to analysis using the chi-square test. To evaluate the associations between various SHR levels and the likelihood of in-hospital and 1-year mortality, we employed multivariate logistic and Cox regression models, respectively, generating odds ratios (ORs) or hazard ratios (HRs) accompanied by their respective 95% confidence intervals (CIs). Additionally, we conducted Kaplan-Meier survival analysis to assess the incidence rates of the primary outcome event within SHR-defined groups, with inter-group disparities assessed via the log-rank test. Furthermore, the relationship between SHR levels and mortality risk was examined using restricted cubic spline (RCS) curves. The reference group for this analysis was defined as the SHR interval with the lowest incidence rate. To evaluate the potential enhancement in the predictive accuracy of adverse outcome events by incorporating SHR into the existing severity of illness scores (including SOFA score, LODS score, SAPS-II, and Charlson score), the area under the curve (AUC) was calculated. Subsequently, the distinct models were compared using the DeLong test.

The multivariate logistic and Cox regression analyses included adjustments for pertinent baseline factors encompassing demographic parameters (age, sex, weight), medical history (hypertension, DM, MI, and CKD), and interventions (history of insulin use, vasopressors, MV). Additionally, subgroup analyses were conducted, stratifying outcomes based on age, sex, the presence of comorbidities (DM, hypertension, acute MI, and CKD), as well as primary interventions (vasopressors and MV). These subgroup analyses were performed using comprehensive regression models that were adjusted for confounding factors.

## Results

### Baseline characteristics

In this study, we included a cohort of 8,978 critically ill patients for comprehensive analysis. The median age of this cohort was 74.2 years (interquartile range: 65.9–82.0), and among them, 5,452 (60.7%) patients were male. Out of the participants, 4,013 (44.7%) had type 2 diabetes, and 2,734 (30.5%) individuals underwent insulin therapy. The participants were stratified into seven distinct groups (group 1–7) based on their SHR levels: < 0.50 (n = 208), 0.50–0.74 (n = 1,086), 0.75–0.99 (n = 3,649), 1.00-1.24 (n = 2,098), 1.25–1.49 (n = 957), 1.50–1.74 (n = 458), and ≥ 1.75 (n = 522). The baseline characteristics of these seven groups are summarized in Table 1. Furthermore, for additional context, Table S1 presents a comparison of baseline characteristics between survivors and non-survivors during the in-hospital period, while Table S2 offers a similar comparison for the 1-year follow-up.

**Table 1:** Baseline characteristics grouped according to SHR levels

Variables	Total	Groups (group 1–7) divided by SHR							P value
		< 0.50	0.50–0.74	0.75–0.99	1.00–1.24	1.25–1.49	1.50–1.74	≥ 1.75	
Sample, %	8978 (100)	208 (2.3)	1086 (12.1)	3649 (40.6)	2098 (23.4)	957 (10.7)	458 (5.1)	522 (5.8)	
Age, year	74.2 (65.9–82.0)	71.1 (62.1–78.9)	74.1 (65.7–82.0)	74.1 (65.9–81.9)	74.7 (66.1–82.4)	75.0 (67.3–82.5)	74.1 (66.6–82.0)	72.6 (63.4–81.1)	< 0.001
Male, %	5452 (60.7)	123 (59.1)	637 (58.7)	2294 (62.9)	1263 (60.2)	574 (60.0)	246 (53.7)	315 (60.3)	0.004
Weight, Kg	82.5 (69.3–97.3)	83.2 (68.0-99.8)	80.4 (67.9–97.3)	82.9 (70.0–97.0)	82.9 (69.5–97.0)	82.0 (68.6–98.3)	81.6 (67.2–95.9)	83.0 (69.7–97.6)	0.297
Severity of Illness									
SOFA score	5 (3–7)	5 (3–8)	5 (3–7)	5 (3–7)	5 (3–7)	5 (3–7)	6 (3–8)	6 (3–9)	< 0.001
SAPS II score	37 (30–45)	38 (31–47)	36 (30–43)	37 (30–45)	37 (31–46)	37 (31–46)	38 (31–47)	40 (33–50)	< 0.001
LODS score	4 (3–7)	5 (3–8)	4 (3–7)	4 (3–6)	4 (3–7)	5 (3–7)	5 (3–8)	5 (3–8)	< 0.001
Charlson score	6 (5–8)	7 (6–9)	6 (5–8)	6 (4–7)	6 (4–8)	6 (5–8)	7 (5–8)	7 (5–9)	< 0.001
First Care Unit									< 0.001
CCU, %	1180 (13.1)	36 (17.3)	158 (14.5)	361 (9.9)	278 (13.3)	135 (14.1)	95 (20.7)	117 (22.4)	
CVICU, %	3802 (42.3)	48 (23.1)	439 (40.4)	1941 (53.2)	850 (40.5)	302 (31.6)	119 (26.0)	103 (19.7)	
MICU, %	1265 (14.1)	50 (24.0)	191 (17.6)	384 (10.5)	267 (12.7)	161 (16.8)	79 (17.2)	133 (25.5)	
SICU, %	1724 (19.2)	58 (27.9)	209 (19.2)	559 (15.3)	444 (21.2)	218 (22.8)	108 (23.6)	128 (24.5)	
Others, %	1007 (11.2)	16 (7.7)	89 (8.2)	404 (11.1)	259 (12.3)	141 (14.7)	57 (12.4)	41 (7.9)	
Vital Signs									
SBP, mmHg	114 (106–125)	115 (105–129)	114 (106–123)	114 (107–123)	115 (107–126)	116 (107–128)	115 (107–129)	114 (106–127)	0.059
Heart rate, bpm	81 (73–91)	83 (71–93)	80 (72–89)	80 (73–88)	81 (73–92)	83 (75–94)	84 (75–95)	83 (72–95)	< 0.001
Temperature, ℃	36.7 (36.5–37.0)	36.7 (36.4–36.9)	36.7 (36.5–36.9)	36.7 (36.5–36.9)	36.7 (36.5–37.0)	36.8 (36.5–37.0)	36.8 (36.6–37.0)	36.8 (36.6–37.0)	< 0.001
RR, bpm	18 (16–21)	18 (16–21)	18 (17–21)	18 (16–20)	19 (17–21)	19 (17–22)	19 (17–21)	20 (17–22)	< 0.001
Comorbidities									
T2DM, %	4013 (44.7)	197 (94.7)	618 (56.9)	1164 (31.9)	876 (41.8)	488 (51.0)	295 (64.4)	375 (71.8)	< 0.001
Hypertension,%	6457 (71.9)	145 (69.7)	771 (71.0)	2629 (72.1)	1533 (73.1)	668 (69.8)	335 (73.1)	376 (72.0)	0.565
AMI, %	1335 (14.9)	40 (19.2)	159 (14.6)	376 (10.3)	328 (15.6)	181 (18.9)	101 (22.1)	150 (28.7)	< 0.001
OMI, %	1977 (22.0)	68 (32.7)	283 (26.1)	712 (19.5)	425 (20.3)	217 (22.7)	132 (28.8)	140 (26.8)	< 0.001
CKD, %	3350 (37.3)	141 (67.8)	501 (46.1)	1153 (31.6)	731 (34.8)	348 (36.4)	224 (48.9)	252 (48.3)	< 0.001
Laboratory tests									
WBC, x 10^9/L	8.7 (6.7–11.8)	8.9 (7.1–11.3)	8.2 (6.5–10.8)	8.5 (6.6–11.4)	8.9 (6.7–12.0)	9.1 (6.8–12.8)	9.1 (7.1–12.9)	9.5 (6.7–14.0)	< 0.001
HGB, g/dL	12.0 (10.6–13.4)	11.6 (10.1–13.1)	11.9 (10.6–13.2)	12.3 (11.0-13.6)	12.0 (10.6–13.4)	11.9 (10.5–13.4)	11.4 (10.0-12.7)	11.6 (10.0–13.0)	< 0.001
SCr, mg/dL	1.0 (0.8–1.3)	1.3 (0.9-2.0)	1.0 (0.8–1.4)	1.0 (0.8–1.2)	1.0 (0.8–1.3)	1.0 (0.8–1.3)	1.1 (0.9–1.5)	1.2 (0.9–1.8)	< 0.001
BUN, mg/dL	19 (15–27)	23 (17–38)	20 (15–29)	18 (14–25)	19 (14–27)	20 (15–28)	21 (16–33)	23 (17–36)	< 0.001
Medical History									
Insulin use, %	2734 (30.5)	154 (74.0)	446 (41.1)	798 (21.9)	569 (27.1)	306 (32.0)	197 (43.0)	264 (50.6)	< 0.001
Vasopressor, %	4393 (48.9)	82 (39.4)	495 (45.6)	1914 (52.5)	972 (46.3)	470 (49.1)	211 (46.1)	249 (47.7)	< 0.001
Diuretics, %	7647 (85.2)	185 (88.9)	945 (87.0)	3158 (86.5)	1731 (82.5)	793 (82.9)	396 (86.5)	439 (84.1)	< 0.001
MV, %	3923 (43.7)	95 (45.7)	495 (45.6)	1502 (41.2)	953 (45.4)	440 (46.0)	204 (44.5)	234 (44.8)	0.012

SHR: stress hyperglycemia ratio; CCU: coronary care unit; CVICU: cardiac vascular intensive care unit; MICU: medical intensive care unit; SICU: surgery intensive care unit; SBP: systolic blood pressure; RR: respiratory rate; T2DM: type 2 diabetes mellitus; CKD: chronic kidney disease; AMI: acute myocardial infarction; WBC: white blood cell count; HGB: hemoglobin; SCr: serum creatinine; BUN: blood urea nitrogen; RRT: renal replacement therapy

### Clinical outcomes

**Fig. 2:**
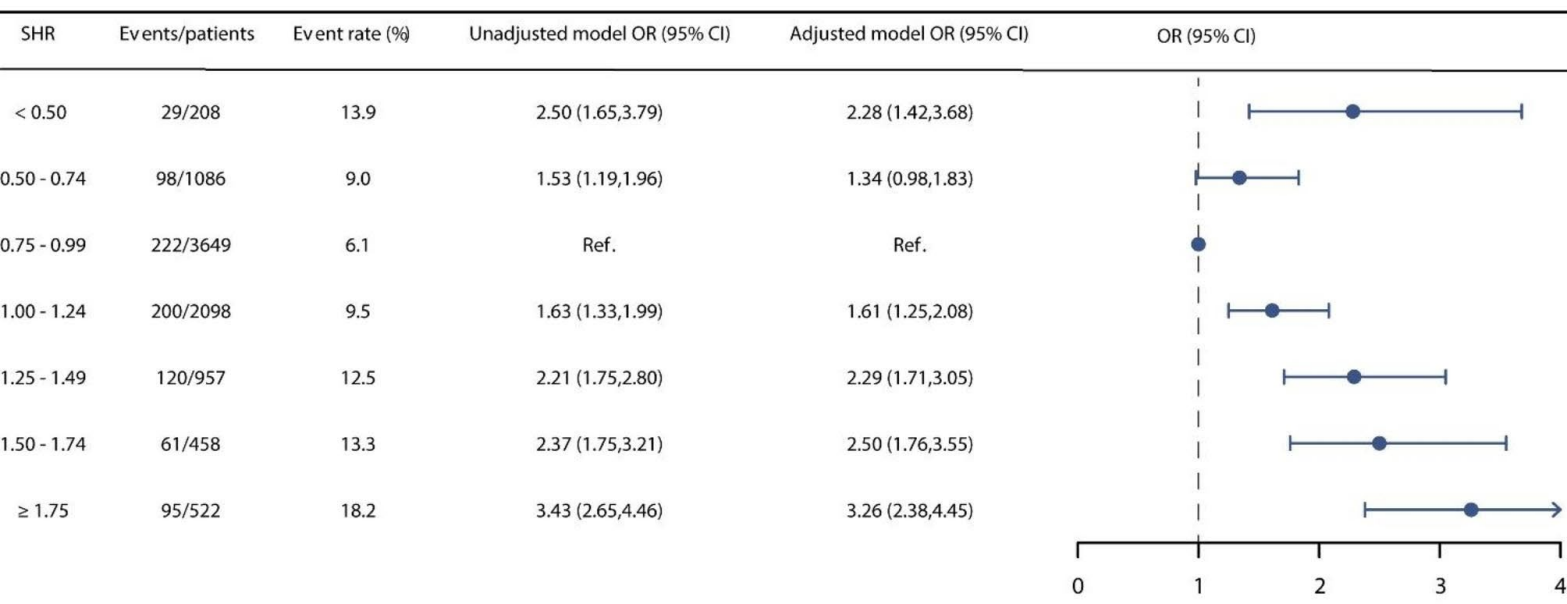
The relationship between SHR and in-hospital mortality

**Fig. 3:**
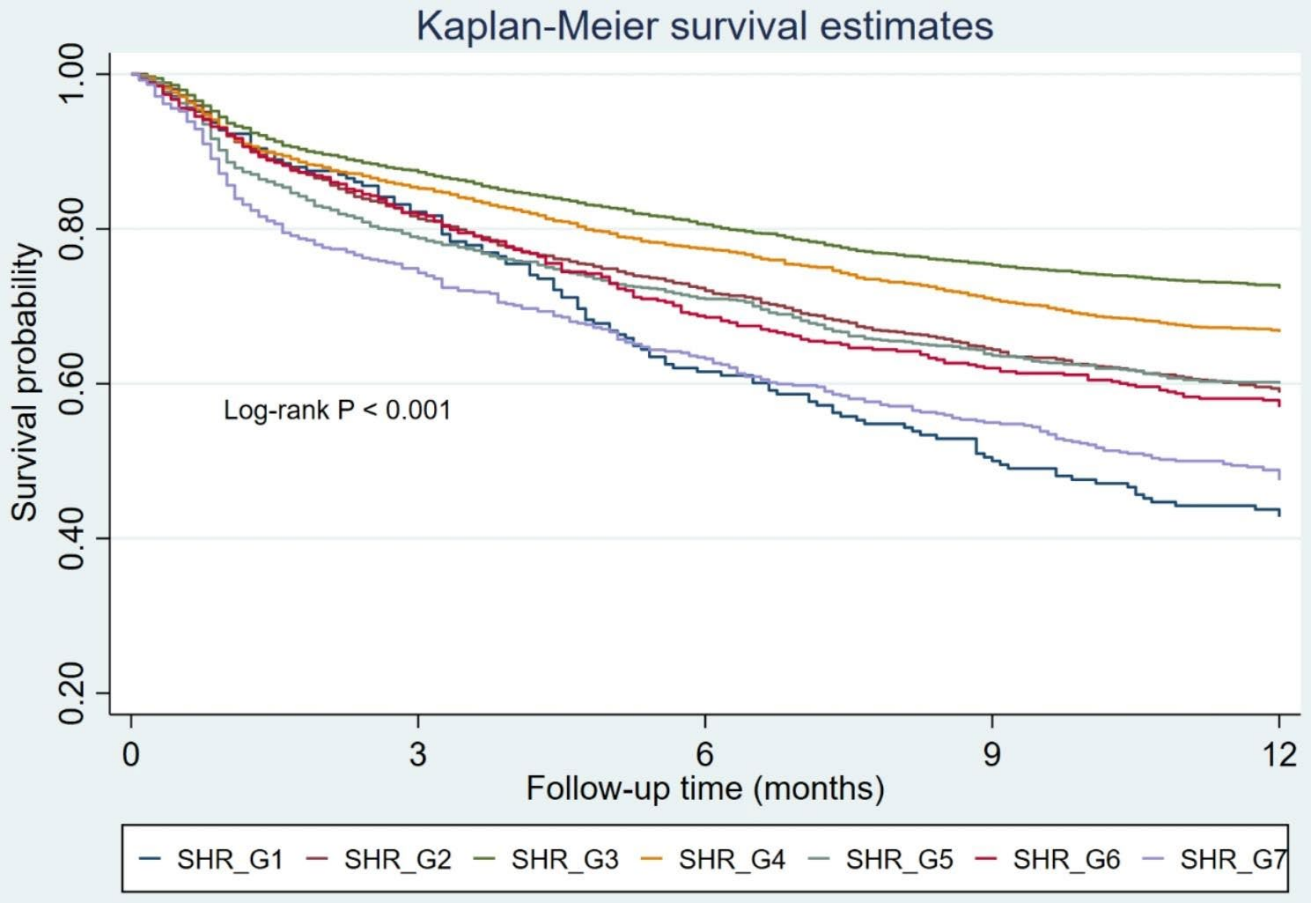
Kaplan–Meier analysis for 1-year mortality based on distinct groups

**Fig. 4:**
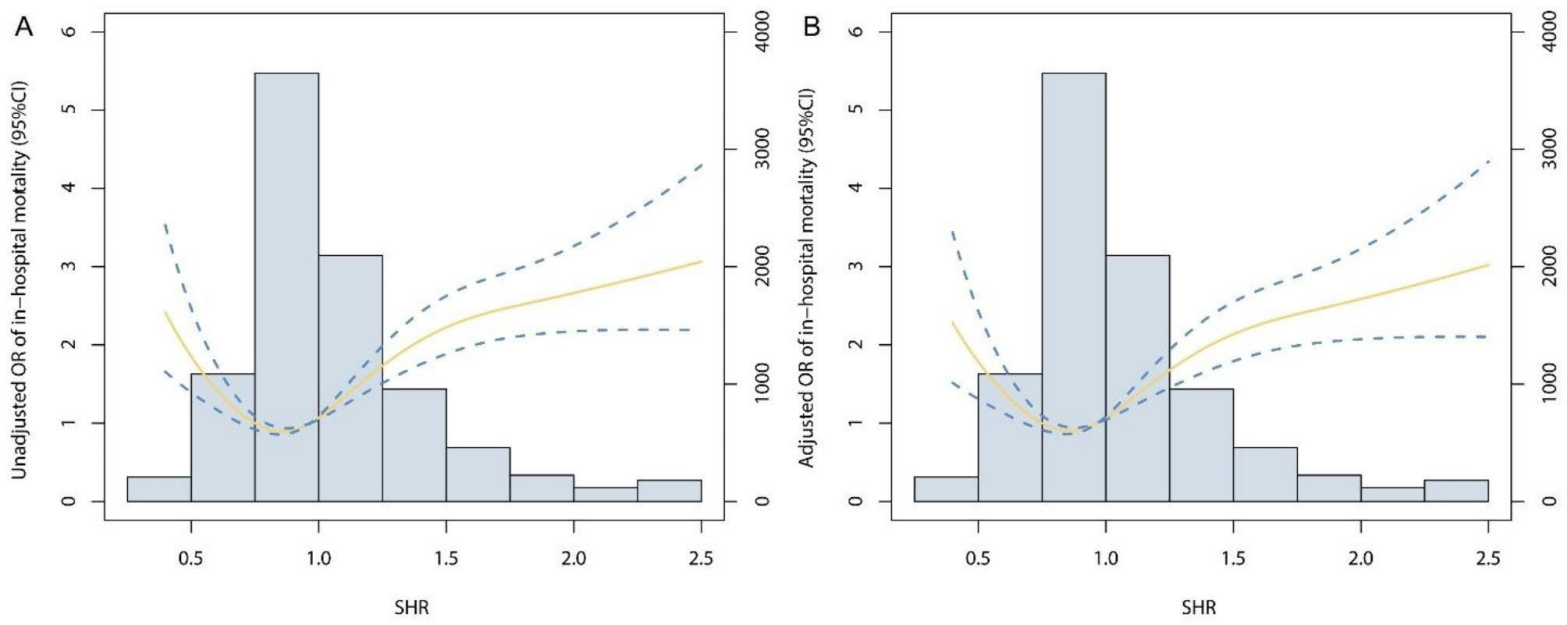
Restricted cubic spline analysis. The U-shaped association between SHR and in-hospital mortality was observed in both (**A**) unadjusted model and (**B**) adjusted model

Throughout the follow-up period, a total of 825 (9.2%) patients experienced in-hospital mortality, and 3,130 (34.9%) individuals succumbed within the span of 1-year follow-up. We stratified patients into seven distinct groups based on SHR levels. Group 3, characterized by the lowest event rate, was set as the reference. It was discerned that Group 1, 2, 4, 5, 6, and 7 exhibited increased risks of in-hospital mortality, featuring unadjusted ORs of 2.50 (95% CI: 1.65–3.79), 1.53 (95% CI: 1.19–1.96), 1.63 (95% CI: 1.33–1.99), 2.21 (95% CI: 1.75–2.80), 2.37 (95% CI: 1.75–3.21), and 3.43 (95% CI: 2.65–4.46), respectively. Within the framework of the multivariable adjusted model, an evident U-shaped correlation between SHR and in-hospital mortality surfaced, yielding ORs of 2.28 (95% CI: 1.42–3.68), 1.34 (95% CI: 0.98–1.83), 1.61 (95% CI: 1.25–2.08), 2.29 (95% CI: 1.71–3.05), 2.50 (95% CI: 1.76–3.55), and 3.26 (95% CI: 2.38–4.45) for Group 1, 2, 4, 5, 6, and 7, respectively (Fig. 2). Upon the stratification of SHR into seven distinct groups, a conspicuous U-shaped correlation between SHR and 1-year mortality was also evident, as graphically depicted in Fig. S1. The Kaplan–Meier curves in Fig. 3 illustrate that patients with an SHR in the range of 0.75–0.99 experienced the lowest 1-year mortality rate (Log-rank P < 0.001). We also performed RCS analysis to further investigate the association between SHR on a continuous scale and mortality. Figure 4 also graphically elucidated the U-shaped trend between SHR and in-hospital mortality, evident in both the unadjusted and adjusted models. The nadir point of this all-cause mortality curve was pinpointed at a SHR level of 0.96, coinciding with the 0.75–0.99 interval (designated as the reference group). Upon meticulous adjustment for multiple covariates, our analysis revealed that for every incremental increment of 0.25 in the SHR level within the 0.75–0.99 interval, the risk of in-hospital mortality escalated by a factor of 1.34 (OR: 1.34, 95% CI: 1.25–1.44). In contrast, for every reduction of 0.25 in the SHR level within the 0.75–0.99 interval, the risk of in-hospital mortality surged by a factor of 1.38 (OR: 1.38, 95% CI: 1.10–1.75). This association was also found between SHR and 1-year mortality (Fig. S2).

Furthermore, we also explored the prognostic utility of SHR in predicting outcomes. Notably, our investigation revealed improvements in the predictive efficacy for in-hospital mortality when SHR was included as a predictive factor alongside established severity scores This enhancement was particularly pronounced in the case of SAPS-II (AUC: 0.726 vs. 0.734, DeLong P = 0.007), SOFA score (AUC: 0.726 vs. 0.734, DeLong P = 0.001), LODS score (AUC: 0.749 vs. 0.757, DeLong P < 0.001), and Charlson score (AUC: 0.649 vs. 0.672, DeLong P < 0.001). This elevated predictive capability remained consistent when forecasting 1-year mortality as well (Table 2).

**Table 2:** Prediction performance of each predictive model for outcomes

Models	AUC (95% CI)	Models	AUC (95% CI)	P for comparison
In-hospital mortality				
SAPS-II	0.726 (0.708–0.744)	+SHR	0.734 (0.717–0.752)	0.007
SOFA score	0.699 (0.680–0.719)	+SHR	0.710 (0.690–0.729)	0.001
LODS score	0.749 (0.730–0.768)	+SHR	0.757 (0.738–0.776)	< 0.001
Charlson score	0.649 (0.631–0.669)	+SHR	0.672 (0.654–0.690)	< 0.001
1-year mortality				
SAPS-II	0.655 (0.643–0.667)	+SHR	0.659 (0.647–0.671)	0.015
SOFA score	0.585 (0.572–0.597)	+SHR	0.595 (0.583–0.607)	< 0.001
LODS score	0.640 (0.628–0.652)	+SHR	0.647 (0.634–0.659)	< 0.001
Charlson score	0.705 (0.694–0.717)	+SHR	0.710 (0.699–0.721)	< 0.001

AUC: area under the curve; other abbreviations are as same as Table 1

### Subgroup analysis

In this study, subgroup analyses were conducted utilizing age, sex, DM, hypertension, AMI, CKD, as well as the application of vasopressors and MV as stratification factors. All outcomes were derived from multivariable regression models. It was ascertained that the U-shaped association between SHR and in-hospital mortality persisted across all examined subgroups. Moreover, we identified noteworthy interactions between SHR and DM. Specifically, patients without DM exhibited a relatively heightened risk of in-hospital mortality when compared to patients with DM (P for interaction = 0.002). Similarly, robust interactions were also discerned in the context of CKD and the use of vasopressors, with P values for interactions amounting to 0.015 and 0.019, respectively (Table 3). Additionally, an analogous approach was employed for the subgroup analysis pertaining to the association between SHR and 1-year mortality, yielding findings that were in line with those observed previously (Table S3).

**Table 3:** Subgroup analysis assessing the association between SHR and in-hospital mortality through odds ratios

Subgroups	Groups divided by SHR							P for interaction
	< 0.50	0.50–0.74	0.75–0.99	1.00–1.24	1.25–1.49	1.50–1.75	≥ 1.75	
Age								0.253
≥ 65	2.13 (1.23–3.67)	1.32 (0.94–1.85)	Ref	1.60 (1.21–2.11)	2.31 (1.69–3.15)	2.50 (1.72–3.63)	2.95 (2.07–4.19)	
< 65	2.46 (0.90–6.76)	1.51 (0.65–3.49)	Ref	1.76 (0.92–3.36)	2.12 (0.96–4.64)	2.27 (0.85–6.08)	4.30 (2.13–8.66)	
Sex								0.119
Male	2.57 (1.39–4.77)	1.47 (0.96–2.26)	Ref	1.82 (1.29–2.57)	2.88 (1.96–4.23)	3.14 (1.96–5.05)	4.09 (2.75–6.16)	
Female	1.82 (0.85–3.88)	1.17 (0.74–1.87)	Ref	1.36 (0.93–1.99)	1.74 (1.12–2.71)	1.90 (1.13–3.19)	2.44 (1.49–3.98)	
DM								0.002
Yes	1.62 (0.98–2.69)	0.99 (0.68–1.45)	Ref	1.11 (0.79–1.55)	1.54 (1.06–2.23)	1.78 (1.18–2.69)	2.22 (1.53–3.21)	
No	10.7 (1.65–68.9)	1.72 (0.98–3.03)	Ref	2.49 (1.69–3.66)	3.77 (2.38–5.96)	4.44 (2.32–8.50)	6.56 (3.63–11.9)	
Hypertension								0.341
Yes	2.25 (1.23–4.11)	1.48 (1.01–2.17)	Ref	1.75 (1.29–2.38)	2.39 (1.68–3.39)	2.82 (1.87–4.25)	3.71 (2.56–5.36)	
No	2.27 (1.03–4.99)	1.09 (0.62–1.90)	Ref	1.35 (0.85–2.15)	2.09 (1.25–3.49)	2.13 (1.08–4.18)	2.52 (1.39–4.54)	
AMI								0.891
Yes	1.44 (0.48–4.27)	0.67 (0.29–1.55)	Ref	0.85 (0.44–1.64)	1.47 (0.73–2.98)	1.66 (0.78–3.52)	2.12 (1.12–4.02)	
No	2.45 (1.44–4.16)	1.46 (1.04–2.05)	Ref	1.74 (1.32–2.29)	2.36 (1.72–3.25)	2.62 (1.76–3.90)	3.32 (2.30–4.79)	
CKD								0.015
Yes	1.80 (1.03–3.14)	0.99 (0.65–1.50)	Ref	1.27 (0.88–1.82)	1.58 (1.03–2.40)	1.70 (1.05–2.73)	2.26 (1.46–3.48)	
No	2.60 (0.98–6.90)	1.78 (1.09–2.89)	Ref	1.98 (1.37–2.84)	3.12 (2.09–4.66)	3.66 (2.19–6.10)	4.77 (3.03–7.49)	
Vasopressor								0.019
Yes	2.91 (1.55–5.46)	1.42 (0.94–2.14)	Ref	1.69 (1.20–2.36)	2.20 (1.50–3.22)	3.35 (2.15–5.21)	4.64 (3.13–6.89)	
No	1.60 (0.75–3.41)	1.14 (0.70–1.86)	Ref	1.44 (0.97–2.12)	2.33 (1.50–3.62)	1.59 (0.87–2.88)	1.84 (1.07–3.16)	
MV								0.104
Yes	1.59 (0.75–3.36)	1.38 (0.88–2.18)	Ref	1.45 (0.97–2.16)	1.96 (1.24–3.10)	1.64 (0.93–2.92)	1.84 (1.08–3.13)	
No	2.82 (1.52–5.34)	1.19 (0.76–1.86)	Ref	1.75 (1.25–2.43)	2.49 (1.72–3.62)	3.15 (2.02–4.91)	4.64 (3.14–6.86)	

The abbreviations are as same as Table 1

Furthermore, it is imperative to note that the SHR value is notably influenced by the preceding glycemic status. Consequently, to comprehensively explore this influence, we conducted RCS analysis in both cohorts of patients with and without DM. Remarkably, our analysis revealed a consistent U-shaped association between SHR and both in-hospital mortality and 1-year mortality outcomes (Fig. S3, S4). This distinctive pattern indicated that the nadir point of 0.96 represented a pivotal juncture. Any deviation of the SHR value from 0.96 was consistently linked with an elevated risk of mortality.

## Discussion

To the best of our understanding, this study represents a significant contribution by demonstrating a U-shaped correlation between SHR and prognosis among critically ill patients. This correlation underscores that both low and high levels of SHR are linked to heightened mortality rates. Furthermore, our investigation reveals SHR’s novelty as a straightforward and effective prognostic tool for mortality prediction. This revelation holds the potential to enhance the predictive accuracy of prevailing severity assessment scores.

Stress hyperglycemia is a prevalent occurrence within critical care environments. Notably, previous studies reported that nearly half of the patients in the ICU displayed stress-induced hyperglycemia [2, 14]. The underlying mechanisms involve the activation of the hypothalamic-pituitary-adrenal (HPA) axis and the sympathoadrenal system [1]. This stress-induced response becomes particularly pronounced within critical care settings. Numerous studies have demonstrated that stress hyperglycemia is an independent risk factor for mortality. Mamtani et al. demonstrated a significant association between stress hyperglycemia and both ICU mortality and an extended duration of ICU stay in a substantial cohort of 739,152 critically ill patients [15]. Badawi et al. reported a robust correlation between stress hyperglycemia and mortality, encompassing diverse glucose metabolic statuses in 194,772 patients admitted in ICU [16].

SHR serves as a valuable metric for attenuating the influence of extended chronic glycemic factors on stress hyperglycemia levels, thereby accurately reflecting the physiological stress response within the body. The investigation of the association between SHR and mortality among critically ill patients remains notably scarce in the existing literature. Recently, Zhang et al. published findings revealing that an elevated SHR stood as an independent risk determinant for ICU mortality among a cohort of 3887 patients (OR: 2.92, 95% CI: 2.14–3.97, P < 0.001) [17]. Notably, their investigation categorized SHR into two distinct groups, utilizing a cut-off value of 1.23, implying an implicit assumption of a linear relationship between SHR and mortality. In contrast, Yang et al. reported an inverted U-shaped correlation between SHR and outcomes in individuals afflicted with acute coronary syndrome [18]. Consistent with these variations, our study similarly identified a U-shaped interrelation between SHR and both in-hospital and 1-year mortality among critically ill patients.

The underlying mechanisms of the U-shaped association of the SHR with morality in critical ill patients remain uncertain and might include the following mechanisms. Stress hyperglycemia is postulated to represent a physiological response aimed at reestablishing homeostasis amidst intense stress. Certain studies have even proposed that mild-to-moderate stress hyperglycemia can serve as a protective factor during times of stress, particularly in the context of ischemia [1]. This phenomenon is underscored by the observation that in animal models of hemorrhagic shock, the administration of a hypertonic glucose solution yielded enhancements in cardiac output, blood pressure, and survival rates [19]. Moreover, stress hyperglycemia holds the capacity to elevate the expression of cell survival factors, including vascular endothelial growth factor and hypoxia-inducible factor-1α. This, in turn, led to a reduction in cell apoptosis, diminished infarction size, and enhancements in cardiac systolic function within a MI rat model [20]. Furthermore, it is noteworthy that moderate stress hyperglycemia, characterized by blood glucose levels ranging from 140 to 220 mg/dL, serves to optimize cellular glucose uptake while concurrently averting hyperosmolarity [21]. It is consistent with our findings that mild to moderate stress hyperglycemia has a protective effect in critical ill patients.

Moreover, our analysis revealed a substantial interaction between glucose metabolic statuses and the connection linking SHR to mortality. Specifically, patients without DM manifested a notably increased risk of in-hospital mortality in contrast to their counterparts with DM. This difference was also reported in previous studies. For instance, in 2012, Kerby et al. put forth that stress hyperglycemia exhibited a strong association with mortality in non-diabetic patients rather than in those with diabetes [22]. Similarly, Wei et al. documented a profound connection between SHR and heightened in-hospital mortality risk among patients with ST-elevation myocardial infarction. Notably, this association was confined to non-diabetic patients in subgroup analysis, even after accounting for confounding variables [23]. This consistent pattern aligns with the observations reported by Zhang et al. [17]. The precise underlying mechanisms remain elusive. Nonetheless, prior research has illuminated those diabetic individuals, due to their sustained presence in a chronic state of inflammation and oxidative stress, might exhibit an adaptive response to the cascade of pathophysiological processes triggered by stress hyperglycemia [24]. Consequently, they could potentially yield comparatively favorable outcomes in the context of adverse consequences associated with stress hyperglycemia, when juxtaposed with non-diabetic patients. Moreover, it is worth noting that diabetic patients undergoing insulin treatment may experience a more robust anti-inflammatory effect, which could further contribute to their relatively better outcomes [25, 26].

### Limitations

While unveiling the significant U-shaped link between SHR and prognosis in critical illness, this study recognizes limitations. Its retrospective design introduces biases and potential uncontrolled factors. Despite adjustments, unmeasured variables could impact outcomes. The study focuses solely on SHR’s connection to mortality, neglecting associations with other indicators. Future research should confirm if this U-shaped pattern applies to endpoints like hospital stay, organ function, and long-term morbidity. While promising, SHR’s practical use as a predictive biomarker needs validation. Prospective studies are vital to confirm if optimizing SHR improves outcomes and complements existing tools.

## Conclusion

In conclusion, this study sheds light on a U-shaped association between SHR and prognosis in critically ill patients, emphasizing the significance of optimal glycemic control in this context. The findings highlight that both low and high SHR levels are linked to increased mortality rates. The inflection point of SHR for poor prognosis was 0.96. Furthermore, SHR emerges as a novel and efficient biomarker for mortality prediction, offering potential to enhance the predictive accuracy of conventional severity scores.

## Electronic supplementary material

Below is the link to the electronic supplementary material.

Supplementary Material 1

## Data Availability

The datasets used during the current study are available from the corresponding author on reasonable request.
