# Correction to: Prognostic significance of the stress hyperglycemia ratio in critically ill patients

**DOI:** 10.1186/s12933-024-02528-0

**Published:** 2024-12-06

**Authors:** Le Li, Minghao Zhao, Zhuxin Zhang, Likun Zhou, Zhenhao Zhang, Yulong Xiong, Zhao Hu, Yan Yao

**Affiliations:** Chinese Academy of Medical Sciences, Peking Union Medical College, National Center for Cardiovascular Diseases, Fu Wai Hospital, Beijing, 100037 China

**Correction to: Cardiovascular Diabetology (2023) 22:275** 10.1186/s12933-023-02005-0

In the original publication of this article [1], the author noticed the error in the funding number.

The incorrect and correct funding information are presented in this correction article.

Incorrect Funding:

This study was supported by Medical and Health Technology Innovation Project of Chinese Academy of Medical Sciences (2021-CXGC09-1).

Correct Funding:

This study was supported by Chinese Academy of Medical Sciences Innovation Fund for Medical Sciences (CIFMS, 2021-I2M-1-063).

## References

[CR1] Li L, Zhao M, Zhang Z, et al. Prognostic significance of the stress hyperglycemia ratio in critically ill patients. Cardiovasc Diabetol. 2023;22(1):275. 10.1186/s12933-023-02005-0. 37833697 10.1186/s12933-023-02005-0 PMC10576399

