# Supplementary material for: Prognostic significance of the stress hyperglycemia ratio in critically ill patients
Source: Cardiovasc Diabetol. 2023 Oct 13;22:275. doi: 10.1186/s12933-023-02005-0 (PMC10576399; doi:10.1186/s12933-023-02005-0)
Supplement: Supplementary file 1 — Supplementary Material 1 [file 12933_2023_2005_MOESM1_ESM.docx]

**Prognostic Significance of the Stress Hyperglycemia Ratio in Critically Ill Patients**

**Supplementary Online Content**

**Table S1.** Baseline characteristics grouped by survival status of in-hospital

**Table S2.** Baseline characteristics grouped by survival status of 1-year

**Table S3.** Subgroup analysis for the association between SHR and 1-year mortality

**Figure S1.** The relationship between SHR and 1-year mortality.

**Figure S2.** Restricted cubic spline analysis. The U-shaped association between SHR and 1-year mortality was observed in both (**A**) unadjusted model and (**B**) adjusted model.

**Figure S3.** Association between SHR and in-hospital mortality depicted by restricted cubic spline curve in patients with **(A**) or without (**B**) diabetes mellitus.

**Figure S4.** Association between SHR and 1-year mortality depicted by restricted cubic spline curve in patients with (**A**) or without (**B**) diabetes mellitus.

| **Table S1.** Baseline characteristics grouped by in-hospital mortality | | | |
| --- | --- | --- | --- |
| Characteristics | Survivor (n = 8153) | Non-survivor (n = 825) | P value |
| Age, year | 73.8 (65.6-81.7) | 78.1 (69.9-85.3) | < 0.001 |
| Male, % | 4997 (61.3) | 455 (55.2) | 0.001 |
| Weight, Kg | 83.1 (70.0-97.7) | 76.4 (63.1-92.8) | < 0.001 |
| Severity of Illness |  |  |  |
| SOFA score | 5 (3-7) | 7 (5-11) | < 0.001 |
| SAPS II score | 36 (30-44) | 47 (37-58) | < 0.001 |
| LODS score | 4 (3-6) | 8 (5-11) | < 0.001 |
| Charlson score | 6 (4-8) | 7 (6-9) | < 0.001 |
| First Care Unit |  |  | < 0.001 |
| CCU, % | 1038 (12.7) | 142 (17.2) |  |
| CVICU, % | 3701 (45.4) | 101 (12.2) |  |
| MICU, % | 1074 (13.2) | 191 (23.2) |  |
| SICU, % | 1455 (17.9) | 269 (32.6) |  |
| Others, % | 885 (10.9) | 122 (14.8) |  |
| Vital Signs |  |  |  |
| SBP, mmHg | 115 (107-125) | 110 (101-126) | < 0.001 |
| Heart rate, bpm | 81 (73-90) | 87 (76-101) | < 0.001 |
| Temperature, ℃ | 36.7 (36.5-36.9) | 36.7 (36.5-37.1) | 0.521 |
| RR, bpm | 18 (16-21) | 21 (18-24) | < 0.001 |
| Comorbidities |  |  |  |
| T2DM, % | 3595 (44.1) | 418 (50.7) | < 0.001 |
| Hypertension,% | 5891 (72.3) | 566 (68.6) | 0.026 |
| AMI, % | 1176 (14.4) | 159 (19.3) | < 0.001 |
| OMI, % | 1791 (22.0) | 186 (22.1) | 0.703 |
| CKD, % | 2954 (36.2) | 396 (48.0) | < 0.001 |
| Laboratory tests |  |  |  |
| WBC, x 10^9/L | 8.7 (6.7-11.7) | 9.0 (6.7-12.6) | 0.051 |
| HGB, g/dL | 12.1 (10.7-13.4) | 11.6 (10.3-12.9) | < 0.001 |
| SCr, mg/dL | 1.0 (0.8-1.3) | 1.1 (0.9-1.6) | < 0.001 |
| BUN, mg/dL | 19 (14-27) | 24 (17-37) | < 0.001 |
| Medical History |  |  |  |
| Insulin use, % | 2365 (29.0) | 369 (44.7) | < 0.001 |
| Vasopressor, % | 3897 (47.8) | 496 (60.1) | < 0.001 |
| Diuretics, % | 6987 (85.7) | 660 (80.0) | < 0.001 |
| MV, % | 3556 (43.6) | 367 (44.5) | 0.632 |

The abbreviations are as same as Table 1

| **Table S2.** Baseline characteristics grouped by 1-year mortality | | | |
| --- | --- | --- | --- |
| Characteristics | Survivor (n = 5848) | Non-survivor (n = 3130) | P value |
| Age, year | 72.1 (64.3-79.8) | 78.4 (69.7-85.7) | < 0.001 |
| Male, % | 3734 (63.9) | 1718 (54.9) | 0.001 |
| Weight, Kg | 85.0 (72.0-99.4) | 76.7 (64.0-92.3) | < 0.001 |
| Severity of Illness |  |  |  |
| SOFA score | 4 (3-7) | 5 (3-8) | < 0.001 |
| SAPS II score | 35 (29-42) | 41 (34-50) | < 0.001 |
| LODS score | 4 (2-6) | 6 (3-8) | < 0.001 |
| Charlson score | 5 (4-7) | 7 (6-9) | < 0.001 |
| First Care Unit |  |  | < 0.001 |
| CCU, % | 640 (10.9) | 540 (17.3) |  |
| CVICU, % | 3284 (56.2) | 518 (16.6) |  |
| MICU, % | 512 (8.8) | 753 (24.1) |  |
| SICU, % | 806 (13.8) | 918 (29.3) |  |
| Others, % | 606 (10.4) | 401 (12.8) |  |
| Vital Signs |  |  |  |
| SBP, mmHg | 114 (107-123) | 116 (105-128) | 0.620 |
| Heart rate, bpm | 80 (73-89) | 83 (73-94) | < 0.001 |
| Temperature, ℃ | 36.7 (36.5-36.9) | 36.8 (36.5-37.0) | 0.142 |
| RR, bpm | 18 (16-20) | 19 (17-22) | < 0.001 |
| Comorbidities |  |  |  |
| T2DM, % | 2296 (39.3) | 1717 (54.9) | < 0.001 |
| Hypertension,% | 4189 (71.2) | 2271 (72.6) | 0.327 |
| AMI, % | 781 (13.4) | 554 (17.7) | < 0.001 |
| OMI, % | 1125 (19.2) | 852 (27.2) | < 0.001 |
| CKD, % | 1604 (27.4) | 1746 (55.8) | < 0.001 |
| Laboratory tests |  |  |  |
| WBC, x 10^9/L | 8.8 (6.8-12.1) | 8.5 (6.4-11.4) | 0.028 |
| HGB, g/dL | 12.4 (11.0-13.7) | 11.4 (10.1-12.7) | < 0.001 |
| SCr, mg/dL | 1.0 (0.8-1.2) | 1.1 (0.9-1.6) | < 0.001 |
| BUN, mg/dL | 18 (14-24) | 23 (17-35) | < 0.001 |
| Medical History |  |  |  |
| Insulin use, % | 1256 (21.5) | 1478 (47.2) | < 0.001 |
| Vasopressor, % | 3145 (53.8) | 1248 (39.9) | < 0.001 |
| Diuretics, % | 4992 (85.4) | 2655 (84.8) | 0.494 |
| MV, % | 2349 (40.2) | 1574 (50.3) | < 0.001 |

The abbreviations are as same as **Table S1**

| **Table S3**. Subgroup analysis assessing the association between SHR and 1-year mortality through hazard ratios. | | | | | | | |  |
| --- | --- | --- | --- | --- | --- | --- | --- | --- |
| Subgroups | Groups divided by SHR | | | | | | | P for interaction |
|  | < 0.50 | 0.50 – 0.74 | 0.75 – 0.99 | 1.00 – 1.24 | 1.25 – 1.49 | 1.50 – 1.75 | ≥ 1.75 |  |
| Age |  |  |  |  |  |  |  | 0.782 |
| ≥ 65 | 1.73 (1.38-2.17) | 1.27 (1.12-1.44) | Ref | 1.12 (1.01-1.24) | 1.40 (1.23-1.60) | 1.34 (1.13-1.58) | 1.77 (1.51-2.07) |  |
| < 65 | 1.71 (1.11-2.62) | 1.54 (1.15-2.06) | Ref | 1.16 (0.89-1.51) | 1.53 (1.11-2.12) | 1.21 (0.78-1.88) | 2.00 (1.45-2.76) |  |
| Sex |  |  |  |  |  |  |  | 0.635 |
| Male | 1.70 (1.30-2.22) | 1.39 (1.19-1.62) | Ref | 1.07 (0.94-1.23) | 1.43 (1.22-1.68) | 1.42 (1.14-1.76) | 1.87 (1.55-2.25) |  |
| Female | 1.79 (1.33-2.43) | 1.22 (1.03-1.45) | Ref | 1.17 (1.01-1.36) | 1.40 (1.17-1.68) | 1.24 (0.99-1.57) | 1.76 (1.42-2.19) |  |
| DM |  |  |  |  |  |  |  | 0.042 |
| Yes | 1.51 (1.22-1.87) | 1.19 (1.02-1.38) | Ref | 0.95 (0.82-1.11) | 1.30 (1.10-1.54) | 1.23 (1.02-1.50) | 1.58 (1.33-1.88) |  |
| No | 6.56 (3.09-13.8) | 1.36 (1.14-1.62) | Ref | 1.26 (1.10-1.44) | 1.47 (1.23-1.75) | 1.38 (1.04-1.82) | 2.39 (1.85-3.09) |  |
| Hypertension |  |  |  |  |  |  |  | 0.522 |
| Yes | 1.75 (1.38-2.22) | 1.33 (1.16-1.52) | Ref | 1.16 (1.04-1.30) | 1.40 (1.21-1.62) | 1.35 (1.13-1.62) | 1.80 (1.52-2.13) |  |
| No | 1.72 (1.18-2.51) | 1.27 (1.03-1.57) | Ref | 1.01 (0.83-1.23) | 1.45 (1.16-1.81) | 1.28 (0.94-1.74) | 1.91 (1.46-2.49) |  |
| AMI |  |  |  |  |  |  |  | 0.153 |
| Yes | 1.71 (1.10-2.65) | 0.96 (0.71-1.29) | Ref | 0.87 (0.67-1.12) | 1.22 (0.92-1.64) | 1.08 (0.76-1.51) | 1.80 (1.36-2.37) |  |
| No | 1.75 (1.39-2.18) | 1.37 (1.21-1.55) | Ref | 1.16 (1.04-1.29) | 1.43 (1.25-1.63) | 1.37 (1.15-1.64) | 1.75 (1.48-2.07) |  |
| CKD |  |  |  |  |  |  |  | < 0.001 |
| Yes | 1.59 (1.27-2.00) | 1.09 (0.94-1.26) | Ref | 0.93 (0.81-1.06) | 1.25 (1.06-1.48) | 1.15 (0.94-1.40) | 1.24 (1.03-1.51) |  |
| No | 1.79 (1.16-2.77) | 1.63 (1.36-1.95) | Ref | 1.34 (1.16-1.54) | 1.57 (1.32-1.88) | 1.60 (1.24-2.07) | 2.92 (2.37-3.58) |  |
| Vasopressor |  |  |  |  |  |  |  | < 0.001 |
| Yes | 2.19 (1.58-3.03) | 1.48 (1.23-1.78) | Ref | 1.21 (1.04-1.42) | 1.53 (1.27-1.84) | 1.70 (1.34-2.15) | 2.22 (1.80-2.74) |  |
| No | 1.52 (1.18-1.96) | 1.19 (1.03-1.38) | Ref | 1.02 (0.90-1.16) | 1.30 (1.11-1.53) | 1.08 (0.87-1.33) | 1.48 (1.22-1.79) |  |
| MV |  |  |  |  |  |  |  | < 0.001 |
| Yes | 1.61 (1.22-2.13) | 1.47 (1.25-1.71) | Ref | 1.06 (0.92-1.22) | 1.29 (1.09-1.53) | 1.13 (0.90-1.43) | 1.59 (1.29-1.95) |  |
| No | 1.90 (1.42-2.53) | 1.16 (0.97-1.37) | Ref | 1.19 (1.04-1.37) | 1.55 (1.31-1.84) | 1.59 (1.29-1.98) | 2.13 (1.75-2.58) |  |

The abbreviations are as same as Table S1.


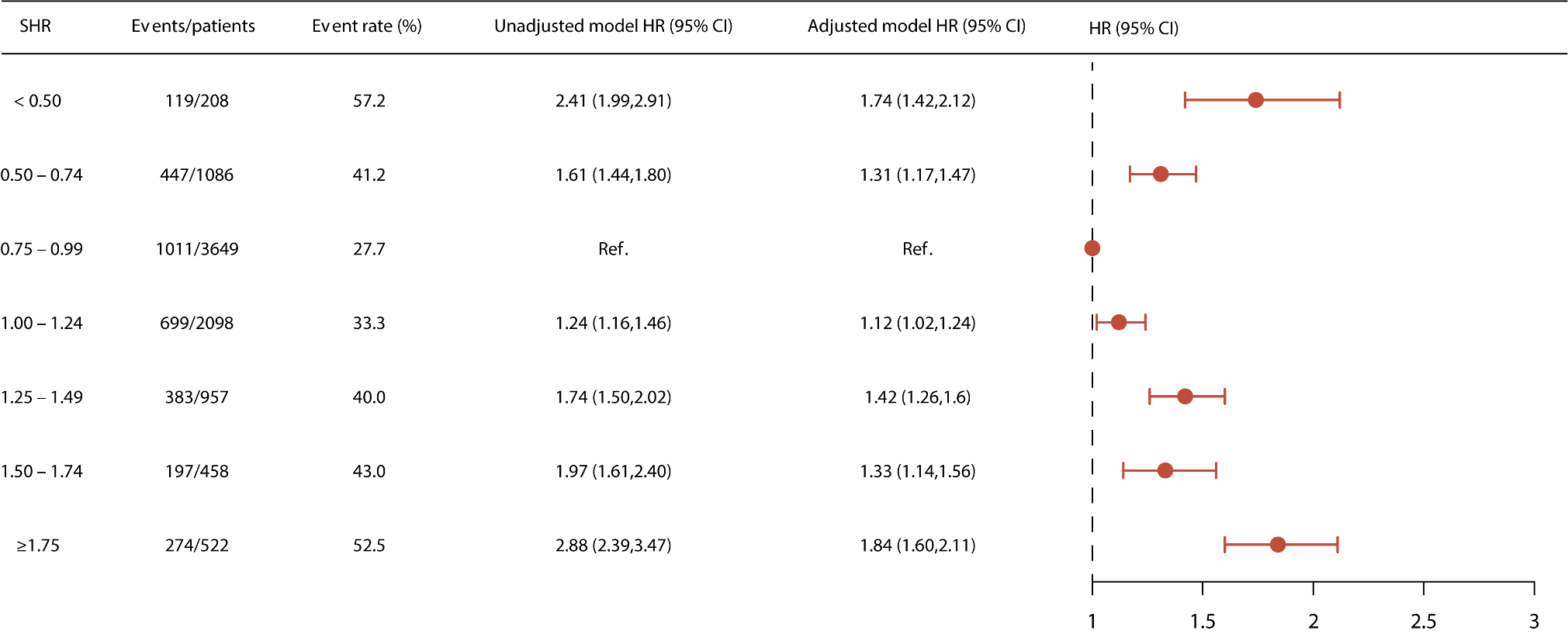
 **Figure S1.** The relationship between SHR and 1-year mortality.


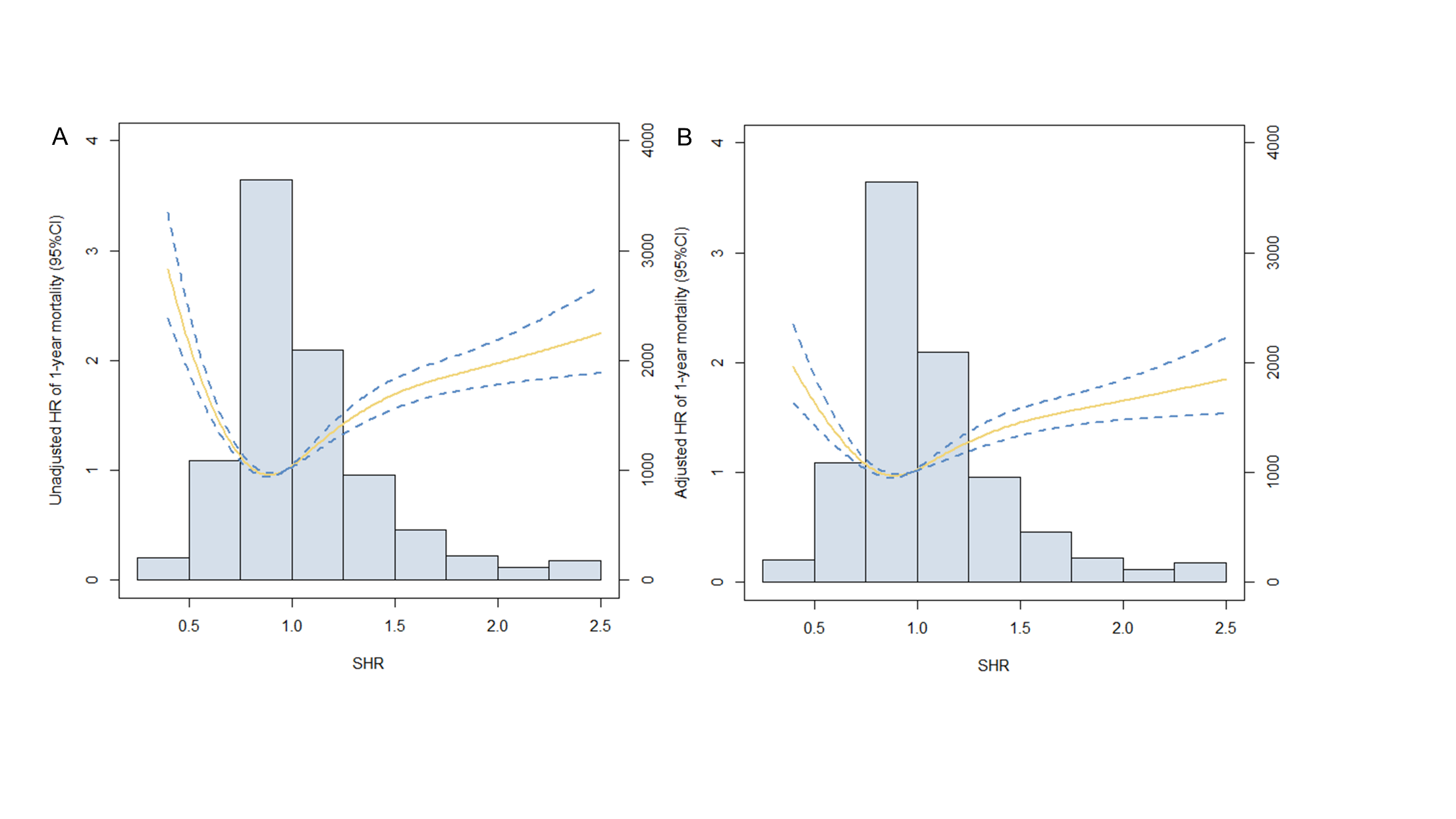
 **Figure S2.** Restricted cubic spline analysis. The U-shaped association between SHR and 1-year mortality was observed in both (**A**) unadjusted model and (**B**) adjusted model.


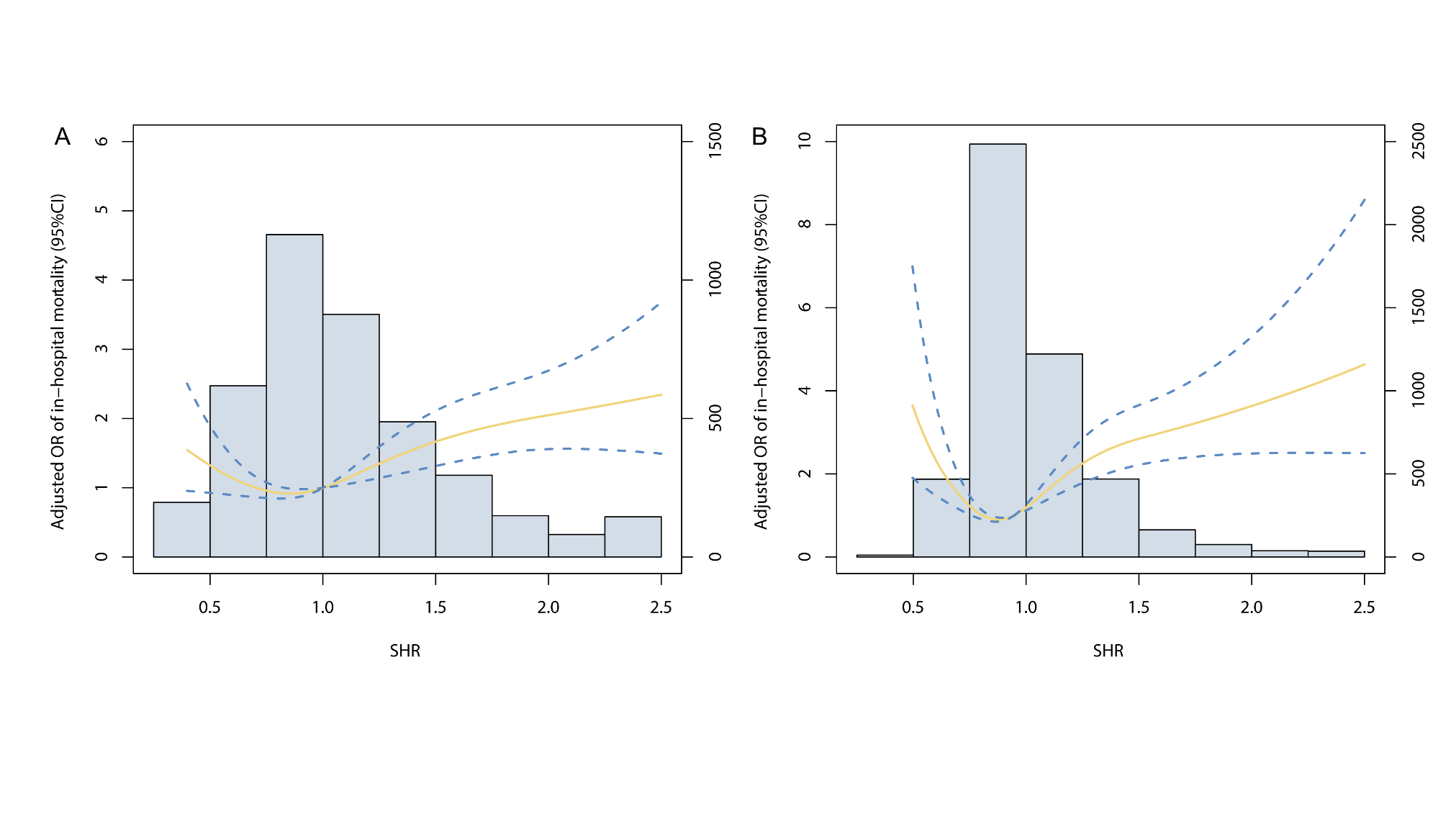


**Figure S3.** Association between SHR and in-hospital mortality depicted by restricted cubic spline curve in patients with (**A**) or without (**B**) diabetes mellitus.


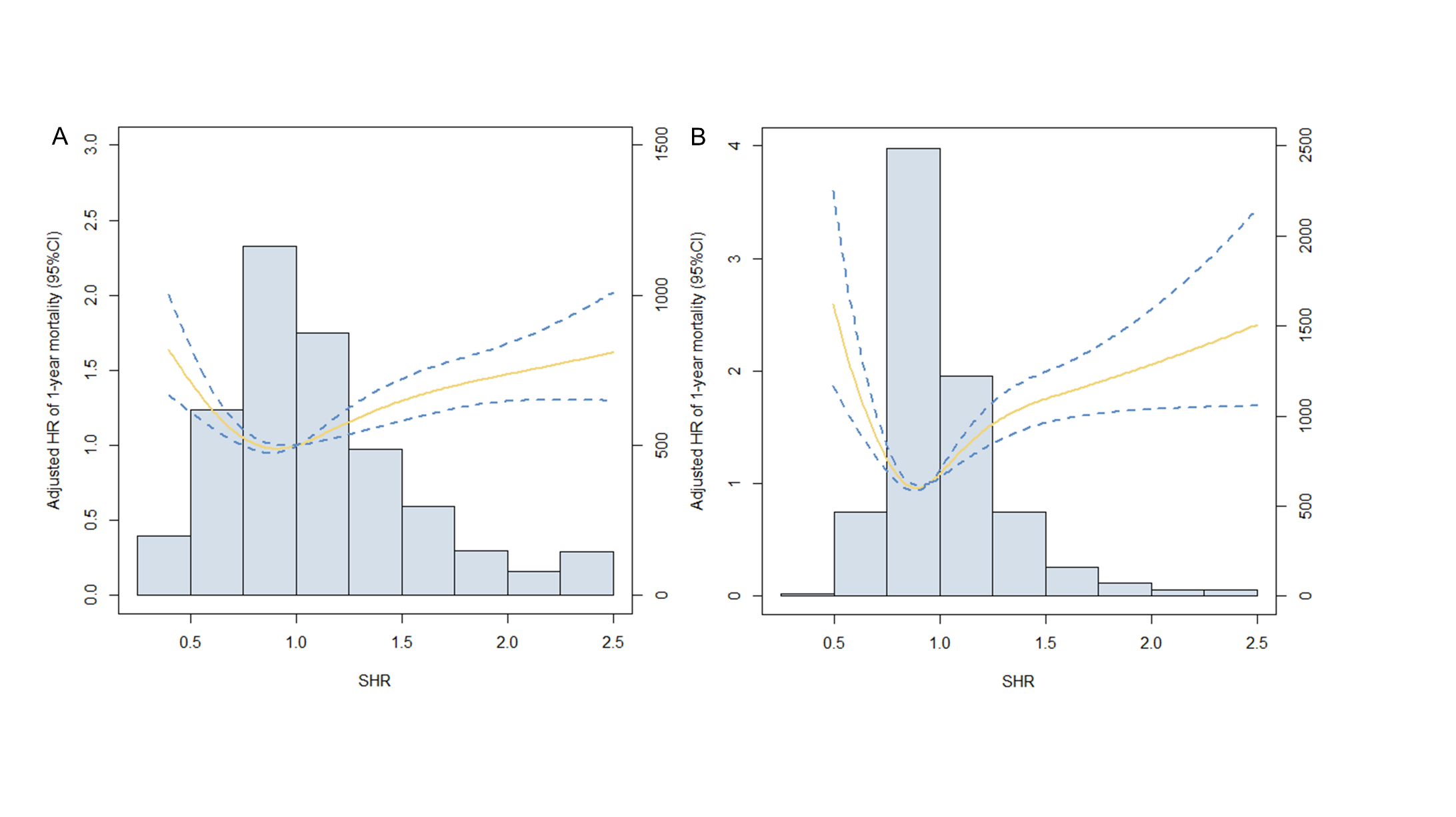


**Figure S4.** Association between SHR and 1-year mortality depicted by restricted cubic spline curve in patients with (**A**) or without (**B**) diabetes mellitus.
